# Fnr Negatively Regulates Prodigiosin Synthesis in *Serratia* sp. ATCC 39006 During Aerobic Fermentation

**DOI:** 10.3389/fmicb.2021.734854

**Published:** 2021-09-17

**Authors:** Di Sun, Xuge Zhou, Cong Liu, Jingrong Zhu, Yunrui Ru, Weijie Liu, Jiawen Liu

**Affiliations:** Jiangsu Key Laboratory of Phylogenomics and Comparative Genomics, School of Life Sciences, Jiangsu Normal University, Xuzhou, China

**Keywords:** *Serratia* sp. ATCC 39006, prodigiosin synthesis, Fnr, aerobic fermentation, transcriptional regulation

## Abstract

The well-known Crp/Fnr family regulator Fnr has long been recognized as an oxygen sensor to regulate multiple biological processes, including the switch between aerobic/anaerobic metabolism, nitrogen fixation, bioluminescence, infection, and virulence. In most cases, Fnr was found to be active under anaerobic conditions. However, its role in aerobic antibiotic metabolism has not yet been revealed. In this research, we report that in the model organism, *Serratia* sp. ATCC 39006, Fnr (Ser39006_013370) negatively regulates prodigiosin production by binding to the spacer between the −10 and −35 region in the promoter of prodigiosin biosynthetic gene cluster under aerobic conditions. Fnr was also shown to modulate the anti-bacterial activity and motility by regulating pathway-specific regulatory genes, indicating that Fnr acts as a global regulator in *Serratia* sp. ATCC 39006. For the first time, we describe that Fnr regulates antibiotic synthesis in the presence of oxygen, which expands the known physiological functions of Fnr and benefits the further investigation of this important transcriptional regulator.

## Introduction

Prodigiosin is a microbial tripyrrole red pigment (Hu et al., 2016), which is a promising reagent with a wide range of bioactivities, including anti-cancer, immunosuppressive, anti-bacterial, and anti-algae activities (Zhang et al., 2016, 2017; Yip et al., 2019; You et al., 2019). Many microorganisms can produce prodigiosin and its derivatives (Williamson et al., 2006; Hu et al., 2016). Originally, the biosynthesis pathway of prodigiosin has been investigated by Williams group who has performed great fundamental work in uncovering the mechanism of prodigiosin production in *Serratia marcescens* Nima (ATCC 29632) and its mutants (Williams and Hussain-Qadri, 1980). Until now, the genetic and biochemical mechanism of prodigiosin synthesis has been revealed in many other *Serratia* strains (i.e., *Serratia* sp. ATCC 39006, *S. marcescens* ATCC 274, and *S. marcescens* PIC 3611) (Williamson et al., 2006; Yip et al., 2019). For the model prodigiosin-producing strain, *Serratia* sp. ATCC 39006, the biosynthetic genes include 15 members, termed *pigABCDEFGHIJKLMNO* (Thomson et al., 2000), and *pigA-pigN* encodes proteins necessary for the production of prodigiosin, which are conserved among *Serratia* sp. strains (Harris et al., 2004; Williamson et al., 2005, 2006). All of these *pig* genes are located in a gene cluster and are transcribed as polycistronic, indicating that the regulation of prodigiosin synthesis can be performed by modulating the transcription of the *pig* gene cluster at the transcription level (Slater et al., 2003; Williamson et al., 2006).

Several regulators have been shown to modulate prodigiosin biosynthesis by binding to the promoter region of the biosynthetic gene cluster in *Serratia* sp., including the gluconate-response GntR-family regulator PigT (Fineran et al., 2005a), the quorum-sensing regulator SmaR (Thomson et al., 2000; Fineran et al., 2005b), the LysR-family regulator HexS (Tanikawa et al., 2006), the SlyA-like regulator Rap, and the Xre-family pleiotropic regulator PigP (Thomson et al., 1997; Slater et al., 2003; Gristwood et al., 2011; Shanks et al., 2013). Some two-component systems, such as PhoB/PhoR, RssA/RssB, and EepS/EepR, also regulate prodigiosin production directly (Gristwood et al., 2009; Horng et al., 2010; Stella et al., 2015). However, the regulatory mechanism of prodigiosin synthesis has not been well established, which limits the genetic engineering of *Serratia* strains to acquire high-yield prodigiosin-producing strains for fermentation.

Fumarate and nitrate reduction regulatory protein (Fnr) belongs to the Crp/Fnr transcriptional regulator family (Mettert and Kiley, 2018). Members of this family regulate target gene expression in the form of a homodimer and bind to the target DNA region by their C-terminal DNA binding domain; this binding ability is controlled by the N-terminal sensor domain in response to various environmental/cellular cues (Green et al., 2001; Korner et al., 2003). *Escherichia coli* Fnr is one of the best-studied members of this family and acts as the primary regulator responding to environmental oxygen concentration (Crack and Le Brun, 2018). The N-terminus of *E. coli* Fnr harbors an oxygen-sensitive [4Fe-4S]^2+^ cluster and forms a transcription-active homodimer in the absence of oxygen. In the presence of oxygen, the [4Fe-4S]^2+^ cluster converts to [2Fe-2S]^2+^, and then the Fnr homodimer dissociates into two monomers, thereby losing the transcription regulatory ability. The [2Fe-2S]^2+^-containing Fnr then loses the iron–sulfur cluster, turning into apo-Fnr when exposed to a high concentration of oxygen or upon extended exposure to oxygen (Khoroshilova et al., 1997; Crack et al., 2008a). Thus, as Fnr is highly sensitive to oxygen, it acts as a pivotal switch between anaerobic metabolism and aerobic metabolism in *E. coli* (Green and Paget, 2004; Crack et al., 2012). Although Fnr homologs also regulate virulence (Green and Baldwin, 1997; Westermark et al., 2000; Marteyn et al., 2010; O’Callaghan et al., 2012; Barbieri et al., 2014), infection ability (Bartolini et al., 2006; Kuntumalla et al., 2011), nitrogen fixation ability, and bioluminescence in several bacteria (Spiro, 1994; Septer et al., 2010; Rutten and Poole, 2019; Shi et al., 2020), knowledge of Fnr in *Serratia* strains remains limited.

In this study, we mutated a prodigiosin high-expressing strain using the mini-*Tn5* transposon. The transposon was found to be inserted into the *ser39006_013370* gene, which encodes a Crp/Fnr-family transcriptional regulator, Fnr. Further investigation found that Fnr binds to the internal region of the −10 region and −35 region of the *pig* gene cluster and represses *pig* gene cluster transcription, thereby downregulating prodigiosin production. We also found that Fnr regulates the anti-bacterial activity and motility of *Serratia* sp. ATCC 39006; thus, Fnr is a global regulator of *Serratia* sp. ATCC 39006. Previous studies have identified Fnr as a primary regulator for bacteria to adapt to the change from an oxic to anoxic environment. Our study reveals the regulatory effect of Fnr on bacterial secondary metabolism under aerobic conditions, which expands its regulatory function, allowing further investigation into the role of Fnr in bacteria.

## Materials and Methods

### Bacterial Strains, Plasmids, Primers, Oligonucleotides, and Culture Conditions

The *E. coli*, *Staphylococcus aureus*, and *Serratia* sp. ATCC 39006 strains and plasmids used in this study are listed in Supplementary Table 1. The primers and oligonucleotides are listed in Supplementary Table 2. All the strains and plasmids constructed in this study have been verified by sequencing. The *E. coli* and *S. aureus* strains were grown in Luria–Bertani broth (LB: yeast extract 5 g/L, tryptone 10 g/L, and NaCl 10 g/L) at 37°C. Wild-type *Serratia* sp. ATCC 39006 and the derived strains were grown at 30°C in LBG medium (10 g/L tryptone, 10 g/L NaCl, 5 g/L yeast extract, and 20 g/L glucose) or M9 medium [2% (w/v) glucose or another carbon source, 2 mM MgSO_4_, 0.1 mM CaCl_2_, 17.1 g/L Na_2_HPO_4_^.^12H_2_O, 3.0 g/L KH_2_PO_4_, 1.0 g/L NH_4_Cl, and 0.5 g/L NaCl]. The *Serratia* sp. ATCC 39006 strains were cultured in 25 mL of liquid medium in 100-mL-volume shake flasks at 200 rpm or on LBG or M9 solid medium supplemented with 1.8% (w/v) agar. Appropriate antibiotics were added to the medium when necessary. The final concentrations of antibiotics are listed as follows: kanamycin (Km), 50 μg/mL; gentamicin (Gm), 15 μg/mL.

### Transposon Mutagenesis and Mapping the Transposon Insertion Sites

To acquire a reporter strain that can be used to detect the *pig* promoter-binding protein, the *lacZ* gene was cloned into the broad-host vector, pBBR1MCS5, resulting in pBBR1MCS5-*lacZ*. The *pig* promoter was inserted upstream of the *lacZ* open reading frame (ORF) to yield the reporter vector pBBR1MCS5-P*_*pig*_-lacZ*. The reporter vector was then transformed into the *Serratia* sp. ATCC 39006 *pigA*-deletion mutant Δ*pigA* (the construction process of Δ*pigA* is shown below) to generate the reporter strain, Δ*pigA*/pBBR1MCS5-P*_*pig*_-lacZ*. Subsequently, transposon mutagenesis was performed as described by Liu et al. (2017), with a slight modification. The mini-Tn5-Km-carrying suicide vector, pRL27, was conjugated to the Δ*pigA*/pBBR1MCS5-P*_*pig*_-lacZ* strain, and the resulting transposon insertion cells were plated on an M9 plate containing 50 μg/mL kanamycin and 15 μg/mL gentamicin and incubated at 30°C. The colonies were inoculated on LB solid medium containing 15 μg/mL gentamicin and X-gal. After incubating at 30°C for 2 days, colonies showing differences in color (more blue or more white) compared to the control strain (Δ*pigA*/pBBR1MCS5-P*_*pig*_-lacZ*) were selected to identify the transposon insertion site. The transposon insertion sites were mapped as described by Liu et al. (2017).

### Gene Manipulation and Complementation

pMMB1, a suicide plasmid for *Serratia* sp. ATCC 39006 gene manipulation, was constructed by replacing the DNA replication origin of pK19*mobsacB* with a π protein-dependent *ori*R6K from pKNG101. To construct an in-frame *fnr* deletion mutant (Δ*fnr*), a 951-bp upstream homologous fragment (−789 to +162 bp relative to the *fnr* start codon) and a 1,032-bp downstream homologous fragment (+700 to +1,731 bp relative to the *fnr* start codon) were amplified using primer pairs WL1003/WL1027 and WL1028/WL1006 separately. The two fragments were digested with *Bam*HI/*Eco*RI and *Eco*RI/*Pst*I, respectively, and ligated to *Bam*HI/*Pst*I-digested pMMB1 to generate an *fnr* in-frame deletion vector, pMMB1-Dfnr. pMMB1-Dfnr was verified by sequencing and then transferred from *E. coli* S17-1(λpir) to *Serratia* sp. ATCC 39006 by conjugation as per the following procedure: Overnight cultured S17-1(λpir)/pMMB1-Dfnr and *Serratia* sp. ATCC 39006 were harvested by centrifugation. The cells were washed twice with sterile LB medium, mixed and dropped onto LB plate, and cultured at 30°C for 12 h. Then, the cells from the lawn were harvested by scraping, washed twice with 10 mM MgSO_4_ buffer, serially diluted, and plated on M9 solid medium containing 50 μg/ml kanamycin. After culturing at 30°C for 2–3 days, the transformants were picked and inoculated into LC medium (yeast extract, 5 g/L; tryptone, 10 g/L), cultured at 30°C and 200 rpm for 12 h, and then serially diluted and plated onto an LCS plate (LC medium supplemented with 200 g/L sucrose). The right Δ*fnr* deletion mutant was verified by colony PCR using primer pairs WL1007 and WL1008, WL1009 and WL1010, and WL1011 and WL1012. A prodigiosin-disrupted strain, Δ*pigA*, was constructed by the same method using primer pairs WL1644 and WL1645, WL1646 and WL1647, WL1648 and WL1649, WL1650 and WL1651, and WL1652 and WL1653.

For complementation of *fnr*, primers WL1301 and WL1302 were used to amplify the DNA fragment containing the ribosome binding site, and the complete ORF of the *fnr* gene. Then, the fragment was digested with *Bam*HI/*Eco*RI and ligated into the *Bam*HI and *Eco*RI sites of pBBR1MCS2-P*_*aacC*__1_* (complementary vector containing a constitutive *aacC1* promoter from pUC-Gm, constructed by our laboratory), resulting in the *fnr*-complemented vector pBBR1MCS2-Cfnr, which was then transformed into Δ*fnr via* conjugation to obtain the complemented strain C*fnr*.

To construct the wild-type (WT)/fnr-FLAG transformant encoding a C-terminal 3 × FLAG-tagged Fnr, the upstream/downstream homologous arm flanking the 3′-terminus region of the *fnr* gene was amplified and cloned into pMMB1. Next, the resulting plasmid was amplified with the primers WL1966 and WL1967 to acquire fragment A, and the oligonucleotide (shown in Supplementary Table 2) encoding 3 × FLAG (DYKDDDDKGDYKDDDDKIDYKDDDDK) was used as a template and amplified by primers WL1592 and WL1963 to acquire fragment B. Finally, the resulting fragments in step two were digested with restriction enzymes, purified, and ligated to obtain the *fnr* C-terminal 3 × FLAG-tag knock-in plasmid, pMMB1-fnr-FlagKin. The plasmid was conjugated into WT to obtain the C-terminal 3 × FLAG-tagged *fnr* transformant WT/*fnr*-FLAG. The C-terminal 3 × FLAG-tagged *pigA* transformants were constructed in the same way, resulting in WT/*pigA-*FLAG and Δ*fnr*/*pigA-*FLAG.

To substitute the constitutive promoter P*_*aacC*__1_* for the native *pig* operon promoter P*_*pig*_*, P*_*pig*_* and its flanking homologous arms were first amplified using the primers WL1979 and WL1980 and subcloned into the pMMB1 vector to generate an intermediate plasmid. Reverse PCR was performed using the intermediate plasmid as a template with the primers WL1981 and WL1982 to acquire the P*_*pig*_*-free part. Second, the *aacC1* promoter region (P*_*aacC*__1_*) from the pUC-Gm plasmid was ligated to the P*_*pig*_*-free part, resulting in the P*_*pig*_*-replacement plasmid, pMMB1-P*_*aacC*__1_*-*pig*. The plasmid was conjugated into WT and Δ*fnr* to replace the *pig* promoter with a constitutive expressing promoter, P*_*aacC*__1_*, resulting in strains WT/P*_*aacC*__1_*-*pig* and Δ*fnr*/P*_*aacC*__1_*-*pig*, respectively.

### Prodigiosin Production and Yield Analysis

Prodigiosin production and yield analysis was performed according to the procedure described by Slater et al. (2003) with slight modifications. To perform fermentation, *Serratia* sp. ATCC 39006 strains stored at −80°C were inoculated into LBG medium and cultured at 30°C, 200 rpm, overnight to obtain activated seed medium. Subsequently, 250 μL seed medium was inoculated into 25 mL fermentation broth (LBG) in 100 mL volume shake flasks and cultured at 30°C, 200 rpm, for 12 h. For prodigiosin yield quantification, 3 mL of fermentation broth was centrifuged at 13,000 *g* for 5 min to harvest cells. The cells were resuspended in 1 mL HCl-acidified methanol (pH 3.0) and placed in a dark room for 30 min to extract prodigiosin. Next, a second centrifugation was performed, and the pellet was discarded; the absorbance of the supernatant at 534 nm (*A*_534_) was measured in a cuvette. The anaerobic fermentation procedure was modified from that of Liu et al. (2020). Briefly, *Serratia* sp. ATCC 39006 that had been cultured overnight was inoculated into 50 mL of LBG medium in 60-mL serum vials. After purging with nitrogen gas for 10 min, the vials were sealed to ensure anoxic conditions. All serum vials were placed at 30°C, 200 rpm, for 12 h before the analysis of growth and prodigiosin production.

### RNA Extraction and Real-Time Reverse Transcription-PCR

*Serratia* sp. ATCC 39006 cells grown in fermentation medium for 12 h were collected for RNA extraction, and total RNA was extracted using the TRNzol universal total RNA extraction agent (Tiangen, China) following the protocol of the manufacturer. Reverse transcription PCR was performed to acquire cDNA, as described by Liu et al. (2017). qRT-PCR assays were performed using SYBR Green qPCR Mix (Biosharp, China), and the results were analyzed using a CFX96 Touch System (Bio-Rad, United States). The qRT-PCR condition was described by Liu et al. (2017), the primers used are listed in Supplementary Table 2, and the *16S* rRNA gene was taken as an internal control. All experiments were performed at least three times.

### Heterologous Expression of His_6_-Tagged *Serratia* sp. ATCC 39006 Fnr Protein

The *Serratia* sp. ATCC 39006 *fnr* encoding sequence was amplified by the primers WL1228 and WL1229. The resulting DNA fragments were inserted into the protein expression plasmid pET-28a(+) to obtain pET28-39006-Fnr. After being screened by DNA sequencing, the vector was transformed into *E. coli* BL21 (DE3) to generate the BL21/pET28-39006-Fnr strain, which was used to heterologously express N-terminal His_6_-tagged Fnr protein. For protein expression and purification, the heterologous protein expression strains were induced by 0.4 mM IPTG and cultured at 16°C, 180 rpm, for 12 h. The broth was centrifuged at 4°C, 11,000 *g*, for 10 min to harvest induced cells, and the cells were washed and resuspended in ice-cold phosphate-buffered saline (PBS) (pH 7.6) buffer containing 10 mM DTT and protease inhibitor (CoWin Biosciences). The cells were then broken in a sonicator (JY88-IIN, Ningbo Scientz Biotechnology Co., Ltd.) on ice, and low-temperature centrifugation was applied to remove the pellet. The supernatant-containing soluble His_6_-39006 Fnr protein was purified by affinity chromatography on Ni-agarose resin (CoWin Biosciences). After washing with washing buffer (ice-cold PBS buffer containing 50 mM imidazole and 10 mM DTT), the target protein was eluted from the resin with elution buffer (ice-cold PBS buffer containing 250 mM imidazole and 10 mM DTT). After verification by SDS-PAGE, the protein was dialyzed in dialysis buffer [20 mM HEPES, pH 7.6, 1 mM EDTA, 10 mM (NH_4_)_2_SO_4_, 10 mM DTT, 30 mM KCl, 0.2% (w/v) Tween-20, and 20% (v/v) glycerol] at 4°C. The concentration of the dialyzed protein was measured using Bradford assay, and the protein was subpackaged and preserved at −80°C.

### Electrophoretic Mobility Shift Assays

Electrophoretic mobility shift assays (EMSAs) were performed as described by Sun et al. (2016). The primers used to amplify the EMSA probes are listed in Supplementary Table 2. The mutated *pig* promoter probe was amplified using primers WL1265 and 1266 (listed in Supplementary Table 2), and the plasmid pMV-P2M was used as template.

### 5′-Rapid Amplification of cDNA Ends

5′-Rapid amplification of cDNA ends (5′-RACE) assays was carried out as described by Sun et al. (2016). The primers used for reverse-transcribed target genes and to identify the transcriptional start site of the target genes are listed in Supplementary Table 2.

### Chromatin Immunoprecipitation

To perform the chromatin immunoprecipitation assay, *Serratia* sp. ATCC 39006 cells were obtained after fermentation for 12 h, and the following steps were performed as described by Zhu et al. (2016), with minor modifications. In brief, *Serratia* sp. ATCC 39006 cells were cross-linked by 1% formaldehyde at 30°C, 150 rpm, for 25 min, and the cross-linking was terminated by the addition of 0.1 M glycine. After washing twice with PBS, the cells were suspended in lysis buffer (50 mM HEPES-Na, pH 8.0, 137 mM NaCl, 1 mM EDTA, and 10 mM Tris–HCl, pH 8.0) and sonicated to shear the genomic DNA to 250–500-bp fragments. The sonicated mixture was centrifuged at 4°C, 13,000 *g*, for 10 min to collect the supernatant. The protein concentration of the supernatant was measured, and lysis buffer was added to 4 mg total protein to a final sample volume of 1 ml. Next, 10 μL of the sample was frozen at −80°C as the input. The rest of the sample was added to 40 μL anti-FLAG affinity gel (Bimake), which was pre-balanced with bovine serum albumin and salmon sperm DNA, and incubated at 4°C for 4 h, with gentle rotation. The gel was then washed, and the protein–nucleic acid complex was eluted. The input and eluate were reverse cross-linked, and the DNA fragments were extracted. DNA from the inputs and samples was dissolved in ddH_2_O for the qPCR analysis. The primers used for the qPCR analysis of the immunoprecipitated DNA are listed in Supplementary Table 2.

### Western Blotting

*Serratia* sp. ATCC 39006 cells grown in liquid medium were harvested and disrupted in a sonicator. After centrifuging at 4°C, 13,000 *g*, for 10 min, the supernatant was collected to measure the protein concentration. The total protein of each sample was adjusted to equal amounts to perform western blotting assays. The western blotting assay was performed as described by Yan et al. (2020), using anti-FLAG-tag mouse monoclonal antibody (CoWin Biosciences) as the primary antibody and HRP-conjugated goat anti-mouse IgG (CoWin Biosciences) as the secondary antibody. An eECL western blot kit (CoWin Biosciences) was used for chemiluminescence detection of the target band.

### Bioassays of Motility, Bacteriostatic Activity, and Exoenzymes

Motility bioassays were performed as described by Fineran et al. (2005b) and Pan et al. (2021), with slight modifications. Overnight cultured *Serratia* sp. ATCC 39006 strains were adjusted to an OD_600_ of 1.0, and 3 μL of medium was spotted onto an LBG plate containing 0.3% agar (for swimming test) or 0.6% agar (for swarming test). The plates were cultured at 30°C for 24 h before examining the halo sizes. An Oxford cup assay was used to determine the bacteriostatic activity, as described by Slater et al. (2003), with slight modification. Carbapenem-sensitive *S. aureus* cells were overlayed on LB medium containing 0.6% agar. Overnight cultured *Serratia* sp. ATCC 39006 medium was adjusted to an OD_600_ of 1.0 and centrifuged. Next, 100 μL filtered supernatant was injected into the Oxford cup, and the plates were incubated at 30°C for 24 h. The diameter of the inhibition zone around the Oxford cup was measured to indicate the anti-bacterial activity. Secreted cellulase and pectinase activities were assessed as described by Liu et al. (2019), with a minor modification. *Serratia* sp. ATCC 39006 strains, adjusted to the same OD_600_ value, were dropped onto M9 plates supplemented with different carbon sources and placed at 30°C for 48 h before the activity test. For the cellulase activity test, 0.5% (w/v) carboxymethylcellulose sodium was supplied. The plate was stained for 30 min using 0.3% Congo red solution, followed by destaining for 30 min with 0.9% NaCl solution. For the pectinase activity test, 0.5% (w/v) pectin was added to the medium. The plate was stained for 5 min with iodine solution containing 3.3 g/L iodine and 6.6 g/L KI, and the plate was washed with water for 1 min. The exoenzyme activity was assessed by measuring the diameter of the clear zone.

## Results

### Fnr Represses *Serratia* sp. ATCC 39006 Prodigiosin Production Under Aerobic Conditions

We performed transposon mutagenesis analysis to identify the direct transcriptional regulator of the *pig* operon. First, a *pigA* in-frame deletion mutant harboring a *pig* promoter-*lacZ* reporter vector (pBBR1MCS5-P*_*pig*_-lacZ*) was constructed to eliminate the interference of the red prodigiosin. Second, transposon insertion mutation was performed, and the transformants were screened on X-gal-containing plates for significantly altered X-gal hydrolysis ability by observing colony color changes. Approximately 5,000 mutants were screened, seven of which had deeper blue colonies and were selected for mapping the transposon insertion site. Two of the insertion mutants with higher X-gal hydrolysis ability (bluer colony) were inserted into two loci in the open reading frame of the *ser39006_013370* gene (Supplementary Figure 1A). This gene was annotated as the Crp/Fnr family transcriptional regulator Fnr, and the predicted amino acid sequence showed a high similarity to *E. coli* Fnr (identity = 94.40%) (Supplementary Figure 1B).

**FIGURE 1:**
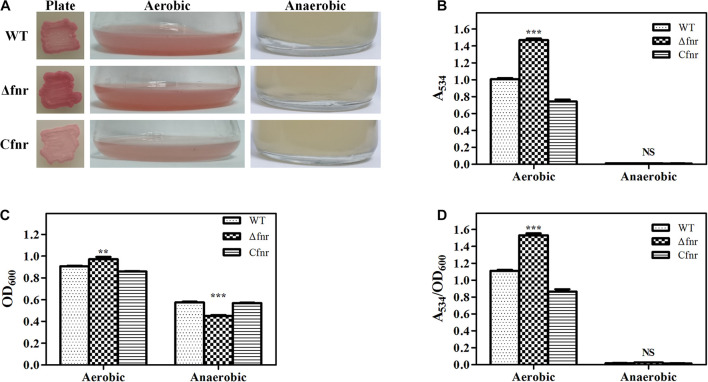
Effect of Fnr on cell growth and prodigiosin production in *Serratia* sp. ATCC 39006. **(A)** Colonies, aerobic fermentation broth, and anaerobic fermentation broth of WT, Δ*fnr*, and C*fnr*. Prodigiosin production **(B)**, cell growth **(C)**, and prodigiosin yield per cell unit **(D)** of WT, Δ*fnr*, and C*fnr* during aerobic and anaerobic fermentation. Experiments were performed at least three times, and data are shown as the mean ± standard deviation (SD). Student’s *t*-test was used to analyze the statistical significance (NS, no significance; **p < 0.01, ***p < 0.001).

The *fnr* in-frame deletion mutant Δ*fnr* and the complementation strain C*fnr* were constructed to identify whether Fnr affected prodigiosin biosynthesis. The plate assay showed that the Δ*fnr* colony was redder than the colony of the WT strain (Figure 1A). Moreover, a higher production of prodigiosin of Δ*fnr* was observed in the shake-flask fermentation assay, in which the prodigiosin yield of Δ*fnr* was 46.2% higher than that of WT (Figure 1B). For both plate assay and the liquid fermentation assays, prodigiosin production was restored in the complementary strain C*fnr* (Figures 1A,B). Although the cell growth in Δ*fnr* was slightly enhanced compared to that in WT (Figure 1C), the prodigiosin yield per cell unit (A_534_/OD_600_) for Δ*fnr* was significantly higher than that for WT (Figure 1D), indicating that the elevation of prodigiosin yield in Δ*fnr* was not due to the change in biomass. Thus, Fnr negatively affects prodigiosin synthesis in *Serratia* sp. ATCC 39006.

Fnr is well recognized as a global transcriptional regulator that is activated under anaerobic conditions, and prodigiosin itself is not made anaerobically (Heinemann et al., 1970). Therefore, we considered whether Fnr is the repressor responsible for inhibiting prodigiosin synthesis under anaerobic conditions. Anaerobic fermentation was performed in the serum vial purged with nitrogen, and all of the examined strains were found to be able to grow under anaerobic conditions (Figure 1C). Similar to WT, no prodigiosin production was detected in Δ*fnr* (Figures 1A,B,D), indicating that prodigiosin synthesis in an anaerobic environment was not suppressed by Fnr. Thus, this report is the first to demonstrate that the deficiency of prodigiosin under anaerobic conditions in *Serratia* sp. ATCC 39006 was not due to Fnr repression and that Fnr was involved in the regulation of prodigiosin synthesis under aerobic conditions.

### Fnr Directly Represses Prodigiosin Biosynthetic Gene Expression at the Transcription Level

**FIGURE 2:**
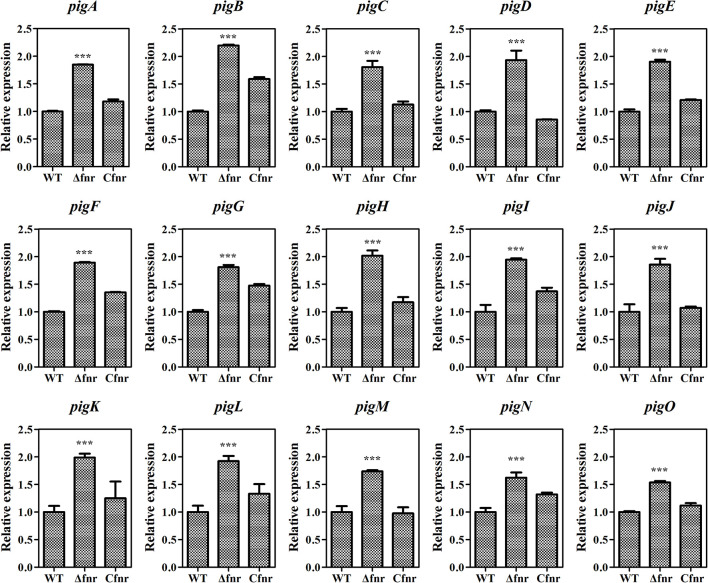
Fnr negatively regulates *pig* gene transcription. The transcription level of the tested genes in WT was assigned as 1, and the transcription levels of tested genes in Δ*fnr* and C*fnr* are displayed as fold changes. The experiments were performed in triplicate, and the data are shown as the mean ± SD. Student’s *t*-test was used to analyze the statistical significance (***p < 0.001).

As Fnr is a transcriptional regulator, it is possible that Fnr represses prodigiosin production by regulating the transcription of the prodigiosin biosynthetic gene cluster. The total RNA of WT, Δ*fnr*, and C*fnr* cells grown in the aerobic fermentation broth was isolated for qRT-PCR, and the transcription levels of 15 *pig* genes arranged in the prodigiosin synthesis operon were measured. The transcription levels of *pig* genes in Δ*fnr* were higher than those in WT, and the transcription was restored in the C*fnr* strain (Figure 2). Meanwhile, the transcription level of the housekeeping gene, *recA*, showed no significant difference in WT, Δ*fnr*, and C*fnr* (Supplementary Figure 2), indicating that the effect of Fnr on the *pig* operon was specific. These results show that the transcription profiles of *pig* genes in WT, Δ*fnr*, and C*fnr* strains were consistent with the prodigiosin yield (Figure 1C), indicating that Fnr represses prodigiosin production *via* negatively regulating the transcription of prodigiosin biosynthetic genes.

**FIGURE 3:**
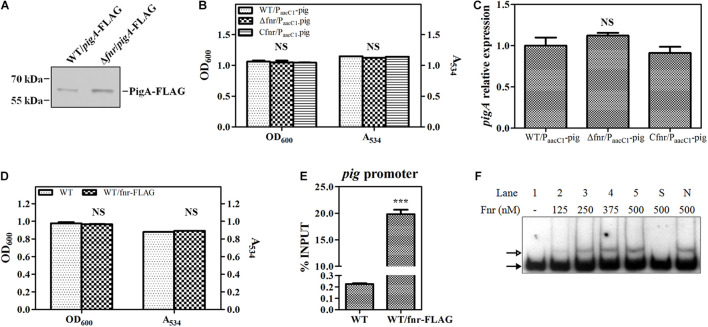
Fnr directly binds to the promoter of the *pig* gene cluster. **(A)** Western blotting analysis of FLAG-labeled PigA in WT and Δ*fnr* strains; 2 μg of total protein was loaded onto the gel. **(B)** Cell growth and prodigiosin production of WT/P*_*aacC*__1_*-*pig*, Δ*fnr*/P*_*aacC*__1_*-*pig*, and C*fnr*/P*_*aacC*__1_*-*pig*. **(C)** Transcription level of *pigA* in WT/P*_*aacC*__1_*-*pig*, Δ*fnr*/P*_*aacC*__1_*-*pig*, and C*fnr*/P*_*aacC*__1_*-*pig* strains. The transcription level of the tested genes in WT/P*_*aacC*__1_*-*pig* was assigned as 1. **(D)** Cell growth and prodigiosin production of WT and WT/fnr-FLAG. **(E)** *In vivo* ChIP-qPCR analysis of Fnr-FLAG binding to the promoter of the *pig* gene cluster. An anti-FLAG affinity gel was used to precipitate the protein-DNA complex, and WT was used as the negative control. The *Y*-axis indicates the relative enrichment value of Fnr on its target site, which was calculated by comparing the Ct value of the sample and input. **(F)** *In vitro* EMSA of His_6_-Fnr binding to the *pig* promoter probe. The concentration of Fnr supplied in each lane is shown, and each lane contained 0.2 nM-labeled DNA probe. Competitive analysis was performed in lane S (supplied with a 100-fold excess of unlabeled specific probe) and lane N (supplied with a 100-fold excess of unlabeled non-specific probe). The solid and hollow arrows indicate the free probe and shifted probe, respectively. Experiments were performed in triplicate, and data are shown in the form of mean ± SD. Student’s *t*-test was used to analyze the statistical significance (NS, no significance; ***p < 0.001).

To confirm this result, a 3 × FLAG tag was knocked into the C-terminus of PigA in WT and Δ*fnr*, resulting in WT/*pigA*-FLAG and Δ*fnr*/*pigA*-FLAG, respectively. Western blotting assay was performed to compare the expression of labeled PigA in these two strains. The results showed that the level of PigA in Δ*fnr*/*pigA*-FLAG was higher than that in WT/*pigA*-FLAG (Figure 3A), which is coincident with the transcriptional analysis (Figure 2), indicating that Fnr regulates *pig* gene expression at the transcription level. We also found that, when the native *pig* promoter was replaced by a constitutive promoter P*_*aacC*__1_* in WT and Δ*fnr*, the prodigiosin yield and the transcription of *pigA* in the resulting strains, WT/P*_*aacC*__1_*-*pig* and Δ*fnr*/P*_*aacC*__1_*-*pig*, were shown to be not significantly different (Figures 3B,C), suggesting that Fnr regulates *pig* gene expression by binding to the *pig* promoter *in vivo*. To verify this hypothesis, a 3 × FLAG tag was knocked into the C-terminus of Fnr, resulting in the strain WT/*fnr-*FLAG, and ChIP-qPCR was performed. Growth and prodigiosin production in WT/*fnr-*FLAG were not significantly different compared to WT, indicating that the knock-in tag does not affect the physiological function of Fnr (Figure 3D). An anti-FLAG monoclonal antibody was used to monitor the binding of FLAG-labeled Fnr to the *pigA* promoter region, and the WT strain was used as the negative control. The enrichment level of Fnr on the *pigA* promoter in WT/*fnr-*FLAG was 87-fold higher than that in the negative control (Figure 3E), indicating that Fnr directly binds to the *pig* promoter *in vivo*. For further confirmation of the binding of Fnr to the *pigA* promoter, His_6_-tagged Fnr was heterologously expressed and purified for an *in vitro* gel shift assay. EMSA experiments found that the recombinant His_6_-Fnr bound to the labeled promoter region of *pigA* (Probe *pigA*) specifically *in vitro*, indicating that Fnr represses *pig* gene transcription directly (Figure 3F). According to the abovementioned results, Fnr represses prodigiosin biosynthesis by directly binding to the promoter region of the *pig* operon and negatively regulates the transcription of *pig* genes.

### Fnr Binds to the Spacer Between the *−*10 Region and the *−*35 Region of the *pig* Gene Cluster

**FIGURE 4:**
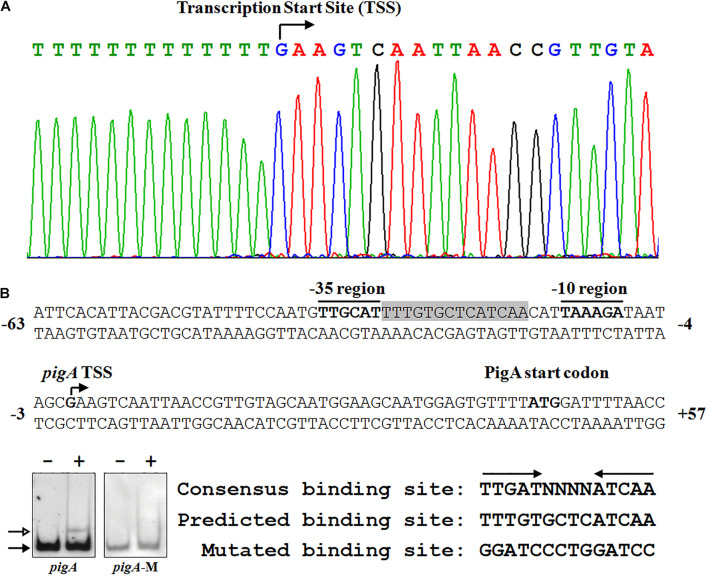
Identification of the Fnr binding site in the *pig* promoter. **(A)** Identification of the *pig* gene transcription start site (TSS) using a 5′-RACE assay (top), and the nucleotide sequence of the *pig* promoter region (bottom). The PigA start codon, *pigA* TSS, and the **–**10 and **–**35 regions are highlighted in bold. The fnr binding site is shown in shadow. The numbers indicate the distance (shown in nt, **–** represents upstream, and + represents downstream) to the TSS (+1). **(B)** Identification of the Fnr binding site. EMSA analysis of the binding between the Fnr and *pig* promoter probes (*pigA*) and the mutant *pig* promoter probe (*pigA*-M) (Left). Comparison between the conserved Fnr binding motif, predicted Fnr binding sites in the *pig* promoter, and the mutated nucleotide sequence (Right). Lane **–**: No protein was added to the reaction mixture. Lane +: 500 nM His_6_-Fnr was added to the reaction mixture. Each lane contains 0.2 nM-labeled DNA probe. The solid and hollow arrows indicate the free probe and shifted probe, respectively.

To clarify the underlying regulatory mechanism of Fnr in prodigiosin synthesis, 5′-RACE was performed to identify the transcription start site (TSS) of *pigA*. The TSS was located at a guanosine 44 nt upstream of the translation start codon, which is coincident with a former study (Slater et al., 2003; Figure 4A). The −10 region and −35 region were also predicted based on the TSS. As the amino acid sequence of *Serratia* sp. ATCC 39006 Fnr is highly similar to that of *E. coli* Fnr (identity = 94.40%) (Supplementary Figure 1B), the conservative binding sequence of *E. coli* Fnr (TTGAT-N_4_-ATCAA) was found in the spacer between the −10 region and −35 region (Figure 4A; Kovacs et al., 2005). To verify whether this sequence is the Fnr binding site, the predicted binding site was mutated (Figure 4B), and the binding activity of Fnr to the probe containing the mutated sequence (Probe *pigA*-M) was evaluated by EMSA. The result shows that Fnr was unable to bind to the mutant probe (Figure 4B), and the Fnr binding site in the *pigA* promoter was identified as the region from 17 to 30 nt upstream of the *pigA* TSS (Figure 4A), indicating that Fnr represses *pig* gene transcription by binding to the spacer between the −10 region and −35 region.

### Fnr Is a Global Regulator in *Serratia* sp. ATCC 39006

**FIGURE 5:**
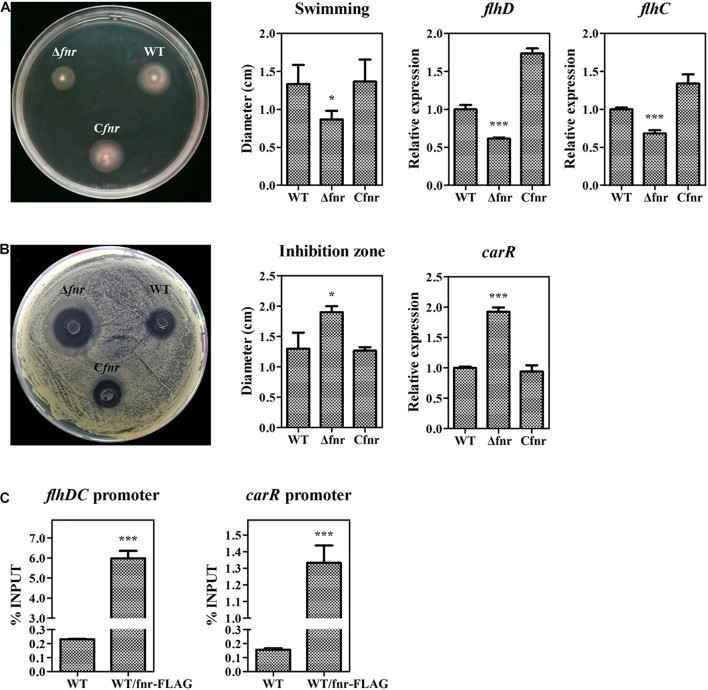
Fnr is a global regulator in *Serratia* sp. ATCC 39006. **(A)** Swimming activity (left), the diameter (cm) of the swimming zone (middle), and the transcription level of *flhD* and *flhC* (right) in WT, Δ*fnr*, and C*fnr*. **(B)** Bacteriostatic activity (left), the diameter (cm) of the inhibition zone (middle), and the transcription level of *carR* (right) in WT, Δ*fnr*, and C*fnr*. **(C)** *In vivo* ChIP-qPCR analysis of Fnr-FLAG binding to the promoters of the *carR* gene and the *flhDC* operon. Anti-FLAG affinity gel was used to precipitate the protein-DNA complex, and WT was used as the negative control. The *Y*-axis indicates the relative enrichment value of Fnr on its target site, which was calculated by comparing the Ct value of the sample and input. The transcription level of the tested genes in WT was assigned as 1. The experiments were performed in triplicate, and the data are shown as the mean ± SD. Student’s *t*-test was used to analyze the statistical significance (*p < 0.05, ***p < 0.001).

In the abovementioned experiments, we have revealed that Fnr represses prodigiosin production by reducing the transcription of *pig* genes. As the Fnr homolog in other bacteria has been reported to be a global regulator modulating various physiological processes, we investigated whether Fnr affects other metabolisms beyond prodigiosin synthesis in *Serratia* sp. ATCC 39006 by plate assay. The swimming ability was reduced in Δ*fnr* but restored in C*fnr* (Figure 5A), indicating that Fnr promotes *Serratia* sp. ATCC 39006 swimming. Given that FlhD (SER39006_RS11350) and FlhC (SER39006_RS11355) were necessary for the motility of *Serratia* sp. ATCC 39006 (Hampton et al., 2016), the transcription levels of *flhD* and *flhC* in WT, Δ*fnr*, and C*fnr* were analyzed. The results showed that the transcription levels of both genes were decreased in Δ*fnr* (Figure 5A), suggesting that Fnr may promote *Serratia* sp. ATCC 39006 swimming ability by increasing the transcription of *flhD* and *flhC.* Besides this, Fnr negatively controlled the bacteriostatic activity of *Serratia* sp. ATCC 39006 (Figure 5B). Transcription analysis showed that *carR* (*ser39006_RS009915*), which encodes the pathway-specific activator of the bacteriostatic carbapenem (McGowan et al., 1995), was elevated in Δ*fnr* (Figure 5B), suggesting that Fnr negatively controls bacteriostatic activity through *carR*. The results of ChIP-qPCR showed that the enrichment of Fnr on the *flhDC* promoter or *carR* promoter was higher (25- and 7.6-fold, respectively) than that in the negative control (Figure 5C), indicating that Fnr regulates the motility and synthesis of carbapenem at the transcriptional level by directly binding to the promoter regions of pathway-specific regulators. Therefore, Fnr acts as a global regulator to control multiple physiological processes in *Serratia* sp. ATCC 39006.

## Discussion

Bacterial secondary metabolism is precisely regulated at different levels, among which transcriptional regulation is the most important for its efficiency and economy. The transcriptional regulation of prodigiosin and other prodiginines has been widely studied (Williamson et al., 2006). In the model undecylprodigiosin-producing strain, *Streptomyces coelicolor*, the transcriptional regulation of undecylprodigiosin is mastered by the cluster-situated regulators, RedD and RedZ (Takano et al., 1992; White and Bibb, 1997; Guthrie et al., 1998). Usually, regulators at higher hierarchies regulate the expression of RedD and RedZ, and the two pathway-specific regulators directly modulate undecylprodigiosin biosynthetic genes (Williamson et al., 2006; Liu et al., 2013). However, no such hierarchical regulatory network was found in the regulation of *Serratia* prodigiosin synthesis. In *Serratia* sp., prodigiosin synthetic genes are arranged in polycistron (Harris et al., 2004; Williamson et al., 2006), indicating that *trans*-acting regulators could directly regulate the whole gene cluster by binding to the promoter upstream of *pigA*, the first gene in the gene cluster. Much of the work on the genetic regulation of prodigiosin production has been performed by the Salmond group, Shanks group, and other researchers. Many regulators, including sigma factors (RpoS) (Wilf and Salmond, 2012), two-component system response regulators (i.e., PhoR/PhoB, RssA/RssB, PigW/PigQ, and EepS/EepR) (Gristwood et al., 2009; Horng et al., 2010; Stella et al., 2015), and transcriptional regulators (i.e., Rap, PigP, PigT, HexS, and SmaR) (Thomson et al., 1997, 2000; Fineran et al., 2005a; Tanikawa et al., 2006; Gristwood et al., 2011; Shanks et al., 2013) have been identified; however, knowledge on the transcriptional regulation of prodigiosin synthesis remains limited.

In the present research, the Crp/Fnr family regulator Fnr (Ser39006_013370) was identified as a negative regulator of *Serratia* sp. ATCC 39006 prodigiosin biosynthesis by directly binding to the target site between the −10 and −35 region of the *pig* gene cluster to repress the transcription of prodigiosin biosynthetic genes. Besides prodigiosin biosynthesis, the Fnr regulon includes carbapenem biosynthetic and flagella genes, suggesting that Fnr is a global regulator in *Serratia* sp. ATCC 39006. The Crp/Fnr family transcriptional regulators usually activate target genes by binding to the target sites upstream of RNA polymerase binding sites and recruiting RNA polymerase (Green et al., 2001). When repressing target gene transcription, the transcriptional regulators of this family usually prevent the formation of an RNA polymerase–DNA complex (Crack et al., 2008b; Bueno et al., 2012). We identified a Fnr binding site located in the spacer between the −10 and −35 region of *pigA* by site-specific sequence mutation (Figure 4B), revealing that Fnr represses *pig* gene expression by blocking RNA polymerase binding to the promoter, which coincides with previous studies.

Fnr and its homologs are involved in the regulation of various metabolisms in many bacteria, and the metabolisms are affected by oxygen concentration. In *E. coli*, Fnr acts as the primary regulator responding to oxygen levels and controls the expression of other regulatory genes (Crack and Le Brun, 2018). Besides this, Fnr homologs (FnrN and FixK) modulate the strict anaerobic process, nitrogen fixation, in rhizobia (Rutten and Poole, 2019). In *Vibrio fischeri*, Fnr regulates bioluminescence under anaerobic conditions but fails to affect luminescence during aerobic culture (Spiro, 1994; Septer et al., 2010). In many pathogenic facultative anaerobes, Fnr senses the oxygen concentration and contributes to optimal growth within the host, promoting survival in the hypoxic environment within macrophages and modulating the virulence and pathogenicity (Green et al., 2014). In summary, previous reports have indicated that, in most bacteria, Fnr regulates its regulon under anaerobic conditions. Prodigiosin production in *Serratia* spp. is a strict oxygen-dependent process (Heinemann et al., 1970). We initially considered whether Fnr is the repressor of prodigiosin synthesis under anaerobic conditions. However, the anaerobic fermentation results showed that this was not the case (Figure 1), indicating that the oxygen-dependent prodigiosin synthesis mechanism is not under the control of Fnr. Therefore, some other unknown regulators may be involved in the inhibition of prodigiosin synthesis under anaerobic conditions, which need further investigation.

Under aerobic conditions, oxygen is the final electron acceptor. When the oxygen concentration decreases, the respiratory mechanism and final electron acceptor change to adapt to the environment (Ravcheev et al., 2007). Such a whole-cell scale metabolism transformation is under precise and strict regulation. In previous studies, the oxygen-sensitive Fnr has been identified as a “switch” to convert bacterial metabolism between anaerobic and aerobic in many bacteria. While in the anaerobic condition Fnr is active and acts as a global regulator, Fnr is inactive and unable to regulate its regulon with oxygen supply. In this research, Fnr was first reported to regulate antibiotic synthesis under aerobic conditions. We also found that Fnr regulates the carbapenem synthesis-specific regulatory gene *carR* and the motility regulatory genes of *Serratia* sp. ATCC 39006 under aerobic conditions. In summary, Fnr acts as a global regulator controlling bacterial physiological processes by modulating the expression of pathway-specific regulators under aerobic conditions, indicating that *Serratia* sp. ATCC 39006 Fnr is active even in the presence of high oxygen concentrations, which is distinct from its homologs. Thus, we speculated that *Serratia* sp. ATCC 39006 employs a particular mechanism to keep Fnr active under aerobic conditions. First, *Serratia* sp. ATCC 39006 Fnr may be less sensitive to oxygen. Although the major iron–sulfur cluster binding residues (Cys20, Cys23, Cys29, and Cys122) were conserved in *Serratia* sp. ATCC 39006 Fnr (Green et al., 1993; Kiley and Beinert, 1998), there is a slight variation in some other N-terminal residues (Supplementary Figure 1B), which may lead to lower oxygen sensitivity. Second, there may have been some cellular anti-oxidant mechanism to protect the Fnr protein in the presence of oxygen. Large amounts of the anti-oxidant, DTT, supplied during the His_6_-Fnr extraction, purification, and dialysis processes, resulted in the transcriptionally active Fnr protein used in the *in vitro* gel shift assay (Figure 3F). This indicates that there may exist a type of redox system to protect the transcription activity of Fnr under aerobic conditions in *Serratia* sp. ATCC 39006 cells. In summary, our investigation expands the physiological function of Fnr, and further investigation will focus on revealing why *Serratia* sp. ATCC 39006 Fnr is active under aerobic conditions. Sato et al. (1998) found that prodigiosin was the H^+^/Cl^–^ symporter that can uncouple oxidative phosphorylation and ATP synthesis. Moreover, work by the Haddix group revealed that prodigiosin production and ATP synthesis could be positively correlated (exponential phase) or negatively correlated (early stationary phase) during aerobic fermentation (Haddix et al., 2008; Haddix and Shanks, 2018, 2020; Haddix, 2021). These findings indicate that prodigiosin is more than a secondary metabolite and that the aerobic respiratory-dependent prodigiosin biosynthesis process is more than a story of transcription and instead is a complex system involving energy supply, respiration, prodigiosin property, and a regulatory network. Thus, investigations into the regulation of prodigiosin synthesis and its physiological function in bacteria have critical biological significance.

## Data Availability Statement

The original contributions presented in the study are included in the article/Supplementary Material, further inquiries can be directed to the corresponding authors.

## Author Contributions

DS contributed to the design of primers and oligonucleotide sequence, RNA extraction, qRT-PCR analysis, ChIP-qPCR, EMSAs, 5′-RACE, western blotting, and writing – original draft preparation. XZ contributed to transposon mutagenesis, fermentation, gene manipulation and complementation experiments, RNA extraction, qRT-PCR analysis, protein expression and purification, EMSAs, 5′-RACE, western blotting, and writing – original draft preparation. CL contributed to the design of primers and oligonucleotide sequence, and gene manipulation and complementation experiments. JZ and YR contributed to transposon mutagenesis and fermentation. WL contributed to the design of the research, western blotting, and reviewing and editing of the manuscript. JL contributed to the design of the research, protein expression and purification, and reviewing and editing the manuscript. All authors contributed to the article and approved the submitted version.

## Conflict of Interest

The authors declare that the research was conducted in the absence of any commercial or financial relationships that could be construed as a potential conflict of interest.

## Publisher’s Note

All claims expressed in this article are solely those of the authors and do not necessarily represent those of their affiliated organizations, or those of the publisher, the editors and the reviewers. Any product that may be evaluated in this article, or claim that may be made by its manufacturer, is not guaranteed or endorsed by the publisher.
