# Supplementary material for: Fnr Negatively Regulates Prodigiosin Synthesis in Serratia sp. ATCC 39006 During Aerobic Fermentation
Source: Front Microbiol. 2021 Sep 17;12:734854. doi: 10.3389/fmicb.2021.734854 (PMC8485047; doi:10.3389/fmicb.2021.734854)
Supplement: Supplementary file 1 [file Data_Sheet_1.docx]

Supplementary Material

# Supplementary Tables

TABLE S1: Strains and plasmids used in this study

TABLE S2: Primers and oligonucleotide used in this study

# Supplementary Figures

FIGURE S1: mini-*Tn5* inserted mutant and amino acid alignment between *Serratia* sp. ATCC 39006 and *E. coli* MG1655

FIGURE S2: The transcription level of *recA* in WT, Δ*fnr* and C*fnr*.

**TABLE S1.** Strains and plasmids used in this study

| **Strain or Plasmid** | **Usage or Description** | **Source or reference** |
| --- | --- | --- |
| ***Escherichia coli* and *Staphylococcus aureus*** | | |
| S17-1(λpir) | Routine transconjugation host and cloning host for pMMB1 plasmid variants. *recA*, *pro*, *hsdR*, *recA*::RP4-2-Tc::Mu, *λpir*, Tmp^R^, Sp^R^, Sm^R^ | Laboratory stock |
| DH5α | Routine cloning host. *fhuA2*, *lacΔU169*, *phoA*, *glnV44*, *Φ80'*, *lacZΔM15*, *gyrA96*, *recA1*, *relA1*, *endA1*, *thi-1*, *hsdR17* | Laboratory stock |
| BL21(DE3) | Protein expression host. *dcm*, *ompT*, *hsdS* (r_B_^-^m_B_^-^) *gal* | Laboratory stock |
| BL21/pET28-Fnr | Heterologous expression of N-terminal His_6_-tagged *Serratia* sp. ATCC 39006 Fnr protein | This study |
| UQ3022 | Donor strain for the mini-Tn*5* transposon containing plasmid, pRL27 | (Larsen et al., 2002) |
| *Staphylococcus aureus* | carbapenem sensitive strain | Laboratory stock |
| ***Serratia* sp. ATCC 39006** | | |
| Wild-type (WT) | EMS induced *lac^-^* derivative of ATCC 39006 | (Thomson et al., 2000) |
| Δ*fnr* | ATCC 39006 Δ*fnr* (*Ser39006_013370*) | This study |
| C*fnr* | Δ*fnr* complemented by pBBR1MCS2-Cfnr | This study |
| WT/*fnr-*FLAG | C-terminal 3×FLAG-tagged *fnr* transformant | This study |
| WT/*pigA*-FLAG | C-terminal 3×FLAG-tagged *pigA* transformant | This study |
| Δ*fnr*/*pigA*-FLAG | C-terminal 3×FLAG-tagged *pigA* in Δ*fnr* | This study |
| WT/P*_aacC1_*-*pig* | replace the promoter region of *pig* operon with *aacC1* promoter | This study |
| Δ*fnr*/P*_aacC1_*-*pig* | replace the promoter region of *pig* operon with *aacC1* promoter in Δ*fnr* | This study |
| C*fnr*/P*_aacC1_*-*pig* | replace the promoter region of *pig* operon with *aacC1* promoter in C*fnr* | This study |
| Δ*pigA*/pBBR1MCS5-P*_pig_-lacZ* | Δ*pigA* harboring a *lacZ* reporter plasmid with a *pigA* promoter | This study |
| **Plasmids** | | |
| pET-28a(+) | Vector for heterologous protein expression in *E. coli*, Km^r^ | Novagen |
| pET28-39006-Fnr | Vector for heterologous expression of N-terminal His_6_-tagged *Serratia* sp. ATCC 39006 Fnr | This study |
| pMV-P2M | Vector containing the nucleotide sequence of EMSA probe pigA2, with its predicted Fnr-binding sites mutated. | Synthesized by BGI  Genomics in this study. |
| pRL27 | Plasmid containing Mini-Tn*5* transposon and a π protein-dependent DNA replication origin (*ori*R6K), Km^r^ | (Larsen et al., 2002) |
| pBBR1MCS2 | Broad-host-range vector, Km^r^ | (Kovach et al., 1995) |
| pBBR1MCS2-P*_aacC1_* | Vector for complementary, Km^r^ | Laboratory stock |
| pBBR1MCS5-P*_pig_*-*lacZ* | Reporter vector for identifying the transcriptional regulators bind to the *pig* promoter | This study |
| pBBR1MCS2-Cfnr | Vector for complement *fnr* in Δ*fnr* mutant | This study |
| pMMB1 | Suicide plasmid for *Serratia* sp. ATCC 39006, replacing the DNA replication origin of pK19*mobsacB* with a π protein-dependent *ori*R6K from pKNG101, *sacB* (modified from *Bacillus subtilis*), *lacZ*, Km^r^ | This study |
| pMMB1-Dfnr | Plasmid used for in-frame deletion of *fnr* gene | This study |
| pMMB1-fnr-FlagKin | Plasmid used to knock in 3×FLAG tag to the C-terminus of *fnr* gene | This study |
| pMMB1-pigA-FlagKin | Plasmid used to knock in 3×FLAG tag to the C-terminus of *pigA* gene | This study |
| pMMB1-P*_aacC1_*-*pig* | Plasmid used to replace P*_pig_* with P*_aacC1_* | This study |

**TABLE S2.** Primers and oligonucleotide used in this study

| **Primer/**  **Oligonucleotide** | **Nucleotide sequence** | **Usage** |
| --- | --- | --- |
| **Primers** | | |
| WL1929 | TCCGCCCTCGAGTGAACCATTAGCGTTCGGATTAAT | Cloning the *pig* promoter |
| WL1930 | TTAAAAGGATCCTCCATAAAACACTCCATTGCTTCC |  |
| WL410 | CAGCAACACCTTCTTCACGA | Mapping the transposon insertion site |
| WL409 | AACAAGCCAGGGATGTAACG |  |
| WL1003 | TTGGATCCGTTTGCAGCCGATTTATG | *fnr* upstream homologous arm |
| WL1027 | CCGGAATTCTTTCTGGATTGGTTTTTTTC |  |
| WL1028 | CCGGAATTCGAAAACAATGAGACGCTG | *fnr* downstream homologous arm |
| WL1006 | CCTACTAGTCAGTGTTACCGATAAAGGC |  |
| WL1007 | ATCAGGCGCAATAAAAGC | Verification of Δ*fnr* deletion mutant |
| WL1008 | GTACCTCAATGAAATCGCTTT |  |
| WL1009 | CTTGCGGAAAAATATGATG |  |
| WL1010 | GTGATCAATAACATGCTCGA |  |
| WL1011 | TTGTTTAAAGCGGGTGAT |  |
| WL1012 | AAGCGACTGATAGTTTCTACC |  |
| WL1644 | TGCCTGCAGCTATCGGTTACTCACGCAACATGG | *pigA* upstream homologous arm |
| WL1645 | TGAGAATTCCTGACTATTTGACAGGTTAAAATCCAT |  |
| WL1646 | AGGGAATTCGGAACATCATCATGATTATTCAAC | *pigA* downstream homologous arm |
| WL1647 | GAAGGATCCATATTATCGAGCAACTCCATATGC |  |
| WL1648 | TAACCATGTTACTGGTAACTGGAA | Verification of Δ*pigA* deletion mutant |
| WL1649 | CACCACAAAGACAGCCATCATCAA |  |
| WL1650 | TCGCAAGAGTTGAAAACGATTGGT |  |
| WL1651 | GACATAGGCAGAGACATCATGAAT |  |
| WL1652 | CCTGATGCAGGTTCCGATATTTAC |  |
| WL1653 | CCAAATCATTGAATCATGGAAAAT |  |
| WL1301 | TGACCAGGATCCATATCAATTATTGCCTGAGC | Complementation of *fnr* |
| WL1302 | TTAAGAGAATTCGTACCTCAATGAAATCGCT |  |
| WL1964 | AATATTGGATCCTCAACATTATCCAAATCGTAGGGC | Construction of pMMB1-fnr-FlagKin plasmid |
| WL1965 | AGGCTGCAGTGATCAATAACATGCTCGACAGTG |  |
| WL1966 | GCTCTAGATAACCTATTGCCGGATCAATGATTTTTCTG |  |
| WL1967 | CCGCTCGAGGGCGTTGCTTTTGGCAGCACCCGCCAGCTC |  |
| WL1592 | CCGCTCGAGGGAGGTGGCGATTACAAGGATGAC |  |
| WL1963 | GCTCTAGAGCCACCTCCTTTATCGTCATCATCTTTGTAGTC |  |
| WL1985 | GGCCGCCTGCAGTGCTTATGGATTTGCACATGGTCC | Construction of pMMB1-pigA-FlagKin plasmid |
| WL1986 | AGGGAAGGATCCATATTATCGAGCAACTCCATATGC |  |
| WL1987 | GGACTAGTAAACTCGGTTTAAGGGGAACATCATCATGA |  |
| WL1988 | CCGCTCGAGACGGGCAATAATCTCTTTCTGGATATCATT |  |
| WL1593 | GGACTAGTTTTATCGTCATCATCTTTGTAGTC |  |
| WL1979 | GGCTATGGATCCCTGTAAAGTGTTTGACCATGCTGT | Construction of the plasmid pMMB1-P*_aacC1_*-*pig* |
| WL1980 | CGCGTACTGCAGCTCGGAAATCAGCAGTTTACTCAT |  |
| WL1981 | ATGGATTTTAACCTGTCAAATAGTCAGTCA |  |
| WL1982 | GGACTAGTAAGGATACCGGTTATTGCACTCACATAGTG |  |
| WL1293 | CAACTTAACAGACCGCCTGCGTGC | qRT-PCR primer for *16S* rRNA |
| WL1294 | GCAGAAGAAGCACCGGCTAACTCC |  |
| WL1279 | TGGAGCAGTGTCGGTTGATGCTCT | qRT-PCR primer for *pigA* |
| WL1280 | GAACCGCGTACTCGGAAATCAGCA |  |
| WL1935 | TGCTGGGTCATTCGTTTGGTGGCT | qRT-PCR primer for *pigB* |
| WL1936 | ATGATCGTCCATCCAGTTGGCCAC |  |
| WL1937 | CAGTGCCATTGCCGTGATTCTACA | qRT-PCR primer for *pigC* |
| WL1938 | CTTATCGGTGCTCCCTGACAGAGG |  |
| WL1281 | ACTGCCCACCGATAAGCGACAGTT | qRT-PCR primer for *pigD* |
| WL1282 | ACTCATCGTCACTGACCGGTCCTA |  |
| WL1939 | GATCAAACCTTCGCTGGTGCCGTA | qRT-PCR primer for *pigE* |
| WL1940 | GGAATTTCCATGTCGTGTGCCAGT |  |
| WL1941 | GGCGGTATCGAAAGCAGTCCTCAG | qRT-PCR primer for *pigF* |
| WL1942 | ACTATCAGTGTACGAGGACCGGAT |  |
| WL1943 | GAGCAAGGTCTCGATTGGCATGCA | qRT-PCR primer for *pigG* |
| WL1944 | AGCCGTTGCACCAGCGCAACCATA |  |
| WL1283 | CGCTGTCAGCGTTGAAGCAGTGTT | qRT-PCR primer for *pigH* |
| WL1284 | GCATCATGGTCAATGGCGGAGTCG |  |
| WL1945 | ATGTGCTGTCCAATCACGCCAGTT | qRT-PCR primer for *pigI* |
| WL1946 | CGCGGCGCAATCTTTCTGTTCGCT |  |
| WL1947 | GACGCAATTGTATGCGCTGAATAG | qRT-PCR primer for *pigJ* |
| WL1948 | TACTGGCAATGATGATCGCCACAC |  |
| WL1285 | GGATTATTGCGCTTGCGAGAGCCT | qRT-PCR primer for *pigK* |
| WL1286 | GGCGTCCATCGAGCTTATGTGAAT |  |
| WL1949 | CAACGGATGGTGAAACAGCGGGCA | qRT-PCR primer for *pigL* |
| WL1950 | CACTCGGCCGATGAGTCAGTGTAA |  |
| WL1951 | TGTCGCTGATCTGGTGGCTTATCA | qRT-PCR primer for *pigM* |
| WL1952 | AATAACGCGTATAGTTGTGCCTGT |  |
| WL1953 | CAATGGGTAGCCATCGTGGTTTAT | qRT-PCR primer for *pigN* |
| WL1954 | CCGGAGCAGAGGATCTGGCCTTTA |  |
| WL1287 | TTGCTACTCGCTCACCAAGACGGC | qRT-PCR primer for *pigO* |
| WL1288 | TTCAGCAGATCCAGACCTCGAACA |  |
| WL2168 | GATCTCGCTGTATTACGGTCCGAA | qRT-PCR primer for *carR* |
| WL2169 | GTCAGGATCGCCAGATTGTTATCA |  |
| WL2172 | ATGCGTTGATGCAACTTACCTTGC | qRT-PCR primer for *flhD* |
| WL2173 | GATCATCAAAGCGGAAATGGCATA |  |
| WL2174 | ACACTGTAGTAGCGTTGAGGCGGT | qRT-PCR primer for *flhC* |
| WL2175 | CGGACTAACGTCCATGCTCGGGTC |  |
| WL2356 | ATGGCTATTGATGAGAACA | qPCR primer for *recA* |
| WL2357 | GATCTTCACCCAATCGCATG |  |
| WL1228 | AAATCTGGATCCATGATCCCGGAAAAGCGAATAATT | Construction of expression vector for His_6_-Fnr |
| WL1229 | CAGAAAAAGCTTAATCATTGATCCGGCAATAGGTTA |  |
| WL1265 | CATGTGTTAATTGTGGGTATG | Amplification of EMSA probe Probe *pigA* or Probe *pigA-*M |
| WL1266 | TATACGCTGACTCATAAATATCTG |  |
| WL2021 | GGTCACTAACGATTCAAGATAT | Identification of *pigA* TSS |
| WL2022 | GCATAATGAAAACGATAATCCAAT |  |
| WL2023 | GACCATGTGCAAATCCATAAG |  |
| WL2180 | GTTACTGGTAACTGGAAAGCTATT | ChIP-qPCR primer for *pigA* |
| WL2181 | GACAGGTTAAAATCCATAAAACAC |  |
| WL2200 | CAACATGCCTATATTACCAACAG | ChIP-qPCR primer for *carR* |
| WL2201 | AACTGAAGTGAGTAAAAGCGAC |  |
| WL2206 | AGGTCGTGTGTAATTGTGGT | ChIP-qPCR primer for *flhDC* |
| WL2207 | CACATACAACGCGATAGTACAT |  |
| **Oligonucleotide** | | |
| 3×FLAG tag | GGAGGTGGCGATTACAAGGATGACGACGATAAGGACTATAA  GGACGATGATGACAAGGACTACAAAGATGATGACGATAAA | Template for cloning 3×FLAG tag sequence |


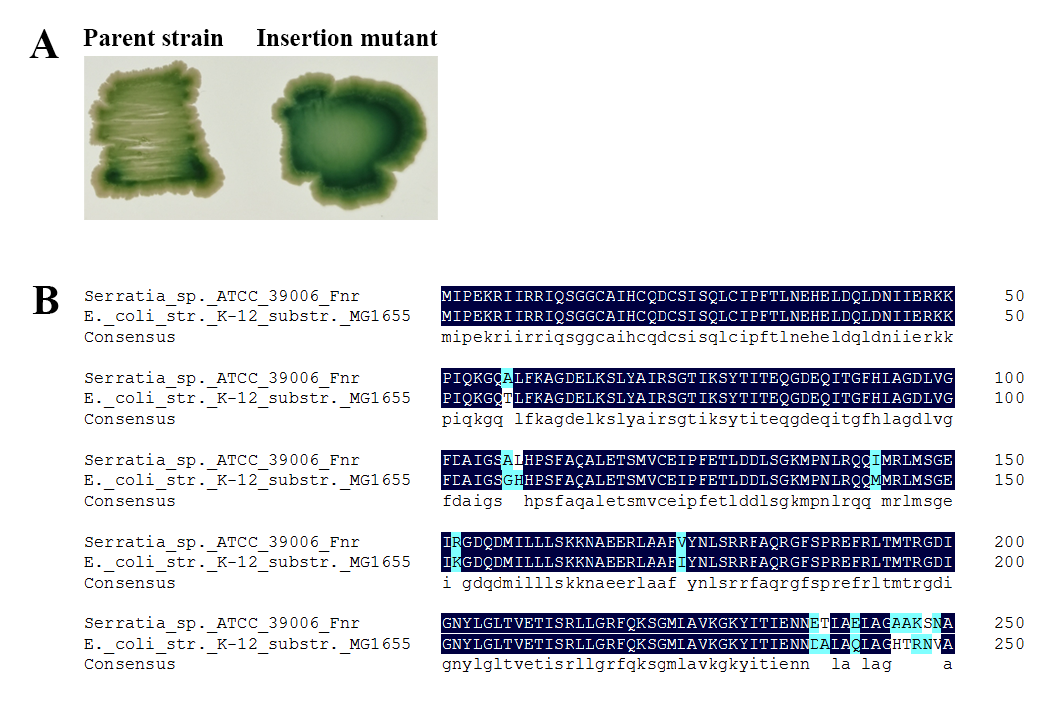


**FIGURE S1.** mini-*Tn5* inserted mutant and amino acid alignment between *Serratia* sp. ATCC 39006 and *E. coli* MG1655. (A) Comparison of parent strain (Δ*pigA*/pBBR1MCS5-P*_pig_-lacZ*) and insertion mutant on X-gal containing plate. (B) Amino acid sequence alignment between *Serratia* sp. ATCC 39006 Fnr and *E. coli* MG1655 Fnr using DNAMAN software.


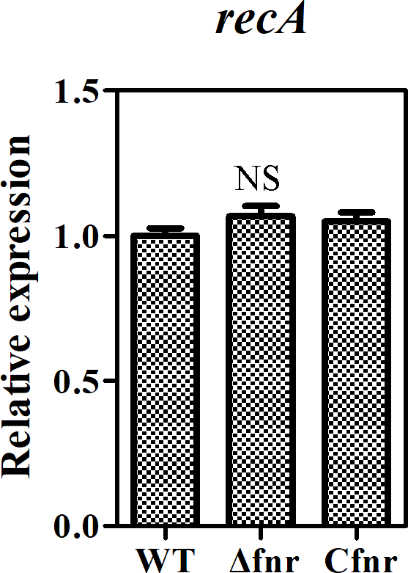


**FIGURE S2.** The transcription level of *recA* in WT, Δ*fnr* and C*fnr*. The transcription level of *recA* in WT was assigned as 1, and the transcription level of tested genes in Δ*fnr* and C*fnr* are displayed as fold changes. Experiments were performed triplicate and data are shown in the form of mean ± SD. Student's *t*-test was used to analyze the statistical significance (NS: No significance).

**REFERENCES**

Kovach, M.E., Elzer, P.H., Hill, D.S., Robertson, G.T., Farris, M.A., Roop, R.M., 2nd, and Peterson, K.M. (1995). Four New Derivatives of the Broad-host-range Cloning Vector pBBR1MCS, Carrying Different Antibiotic-resistance Cassettes. *Gene* 166**,** 175-176. doi: 10.1016/0378-1119(95)00584-1.

Larsen, R.A., Wilson, M.M., Guss, A.M., and Metcalf, W.W. (2002). Genetic Analysis of Pigment Biosynthesis in *Xanthobacter autotrophicus* Py2 Using a New, Highly Efficient Transposon Mutagenesis System that Is Functional in a Wide Variety of Bacteria. *Arch Microbiol* 178**,** 193-201. doi: 10.1007/s00203-002-0442-2.

Thomson, N.R., Crow, M.A., Mcgowan, S.J., Cox, A., and Salmond, G.P. (2000). Biosynthesis of Carbapenem Antibiotic and Prodigiosin Pigment in *Serratia* Is Under Quorum Sensing Control. *Mol Microbiol* 36**,** 539-556. doi: 10.1046/j.1365-2958.2000.01872.x.
